# *NAT2* genotype guided regimen reduces isoniazid-induced liver injury and early treatment failure in the 6-month four-drug standard treatment of tuberculosis: A randomized controlled trial for pharmacogenetics-based therapy

**DOI:** 10.1007/s00228-012-1429-9

**Published:** 2012-11-14

**Authors:** Junichi Azuma, Masako Ohno, Ryuji Kubota, Soichiro Yokota, Takayuki Nagai, Kazunari Tsuyuguchi, Yasuhisa Okuda, Tetsuya Takashima, Sayaka Kamimura, Yasushi Fujio, Ichiro Kawase, Pharmacogenetics-based tuberculosis therapy research group

**Affiliations:** 1 Clinical Pharmacology and Pharmacogenomics, School of Pharmacy, Hyogo University of Health Sciences, Kobe, Hyogo Japan; 2 Clinical Pharmacology and Pharmacogenomics, Graduate School of Pharmaceutical Sciences, Osaka University, Suita, Osaka Japan; 3 Clinical Pharmaceutics and Therapeutics, School of Pharmacy, Hyogo University of Health Sciences, Kobe, Hyogo Japan; 4 National Hospital Organization Toneyama National Hospital, Toyonaka, Osaka Japan; 5 Osaka Prefectural Medical Center for Respiratory and Allergic Diseases, Habikino, Osaka Japan; 6 National Hospital Organization Kinki-Chuo Chest Medical Center, Sakai, Osaka Japan; 7 Japan Anti-Tuberculosis Association Osaka Hospital, Neyagawa, Osaka Japan

**Keywords:** Arylamine *N*-acetyltransferase 2 (NAT2), Isoniazid, Pulmonary tuberculosis, Pharmacogenomics, Randomized clinical trial

## Abstract

**Objective:**

This study is a pharmacogenetic clinical trial designed to clarify whether the *N*-acetyltransferase 2 gene (*NAT2*) genotype-guided dosing of isoniazid improves the tolerability and efficacy of the 6-month four-drug standard regimen for newly diagnosed pulmonary tuberculosis.

**Methods:**

In a multicenter, parallel, randomized, and controlled trial with a PROBE design, patients were assigned to either conventional standard treatment (STD-treatment: approx. 5 mg/kg of isoniazid for all) or *NAT2* genotype-guided treatment (PGx-treatment: approx. 7.5 mg/kg for patients homozygous for NAT2*4: rapid acetylators; 5 mg/kg, patients heterozygous for NAT2*4: intermediate acetylators; 2.5 mg/kg, patients without NAT2*4: slow acetylators). The primary outcome included incidences of 1) isoniazid-related liver injury (INH-DILI) during the first 8 weeks of therapy, and 2) early treatment failure as indicated by a persistent positive culture or no improvement in chest radiographs at the8th week.

**Results:**

One hundred and seventy-two Japanese patients (slow acetylators, 9.3 %; rapid acetylators, 53.5 %) were enrolled in this trial. In the intention-to-treat (ITT) analysis, INH-DILI occurred in 78 % of the slow acetylators in the STD-treatment, while none of the slow acetylators in the PGx-treatment experienced either INH-DILI or early treatment failure. Among the rapid acetylators, early treatment failure was observed with a significantly lower incidence rate in the PGx-treatment than in the STD-treatment (15.0 % vs. 38 %). Thus, the *NAT2* genotype-guided regimen resulted in much lower incidences of unfavorable events, INH-DILI or early treatment failure, than the conventional standard regimen.

**Conclusion:**

Our results clearly indicate a great potential of the *NAT2* genotype-guided dosing stratification of isoniazid in chemotherapy for tuberculosis.

## Introduction

The annual incidence of tuberculosis is still over nine million cases worldwide [1]. With supervision by The Stop TB Strategy, the World Health Organization (WHO) has pursued standardized treatments [2]. The most effective treatment has been a standard 6-month four-drug regimen for the initial treatment of drug-susceptible pulmonary tuberculosis [3, 4]. However, several drug-related adverse reactions can result in discontinuation of the treatment [4, 5]. There can also be considerable morbidity, even mortality, particularly with drug-induced liver injury (DILI) [6–8]. These events incur substantial additional costs because of the increased frequency of outpatient visits, laboratory tests, and hospitalization in more serious instances. Alternative agents can have greater problems with toxicity and are often less effective, and treatment could be prolonged despite attendant challenges to ensure compliance. As a result, the risk of treatment failure and relapse would become even higher. Therefore, individualized drug management using four standard drugs to minimize the incidence of adverse drug reactions is an unmet need in the present clinical circumstances.

Isoniazid (INH), a key drug of the currently recommended regimen for patients with tuberculosis, is metabolized by genetically polymorphic *N*-acetyltransferase 2 (NAT2) [9–11]. The rate of elimination of INH is trimodally distributed in accordance with NAT2 metabolic activity [12, 13]; NAT2 activity status is genetically controlled and basically depends on the number of active alleles (NAT2*4 and *12). Previous findings indicate that a genetic polymorphism is associated with large interindividual differences in the toxicity and efficacy of INH [13–19]. Slow acetylators, patients without any active alleles, develop hepatotoxicity more often than rapid acetylators, patients with two active alleles, during treatment for tuberculosis with the standard regimen [18, 19]. In contrast, rapid acetylators are prone to treatment failure with the standard regimen [13–16], probably due to insufficient exposure to INH. These observations imply that current dosage of INH recommended internationally is too much for slow acetylators and insufficient for rapid acetylators. Thus, we should assume that pharmacogenetically stratified treatment has the potential to avoid unfavorable outcomes with improvements in the cure rate.

Before the start of a prospective clinical trial with the *NAT2* genotype-guided regimen, dose-finding pharmacokinetic studies were independently carried out in Europe and Japan to expect appropriate clinical dosages of INH for each *NAT2* genotype [20, 21]. Based on pharmacokinetic parameters estimated from these dose-finding studies, a genotype-based dosage for the present study was determined as follows; half the standard INH dose for slow acetylators, 1.5 times the standard dose for rapid acetylators, and the standard dose for intermediate acetylators.

Here, we report outcomes of a randomized controlled trial for pharmacogenetics-based tuberculosis therapy (RT-PGTT) conducted in Japan (ClinicalTrials.gov Identifier: NCT 00298870). The purpose of this study was to elucidate whether stratified medicine based on the *NAT2* gene polymorphism could improve the tolerability and efficacy of multidrug therapy for pulmonary tuberculosis with INH.

## Methods

### Study design

This study was designed as a multicenter, two-treatment, parallel group, prospective, randomized, open-label, blinded-endpoint (PROBE) controlled trial, comparing NAT2 genotype-guided and standard dosing of isoniazid in the intensive phase of the 6-month four-drug standard treatment for patients with newly diagnosed pulmonary tuberculosis. This study was approved by the Institutional Review Boards of Osaka University and the participating hospitals, and has been registered on ClinicalTrials.gov. The participating hospitals were National Hospital Organization Toneyama National Hospital, Osaka Prefectural Hospital Organization Osaka Prefectural Medical Center for Respiratory and Allergic Diseases, National Hospital Organization Kinki-Chuo Chest Medical Center, and Japan Anti-Tuberculosis Association Osaka Hospital, all in Osaka, Japan. This study was conducted according to the principles of the Declaration of Helsinki and in compliance with applicable regulatory requests. All of the patients being tested gave written informed consent before the study.

### Study population

The eligible population was identified from a series of patients newly diagnosed with pulmonary tuberculosis (men and women, 20–75 years old), requiring the 6-month four-drug standard treatment for the first time. The exclusion criteria in the pre-study screening phase were as follows: abnormal test results for liver and kidney function (serum aspartate aminotransferase; AST > 45 (IU/L), alanine aminotransferase; ALT > 50 (IU/L), alkaline phosphatise; ALP > 444 (IU/L), total bilirubin > 1.6 (mg/dL), and creatinine > 1.4 (mg/dL)) before the study treatments commenced, long-term use of steroids and/or immunosuppressants, inadequate clinical conditions such as hyperglycemia diabetes mellitus, acute life-threatening chronic progressive disease, pregnancy or lactation, and alcoholism. Patients not expected to complete the study protocol for social reasons were not recruited.

**Fig. 1:**
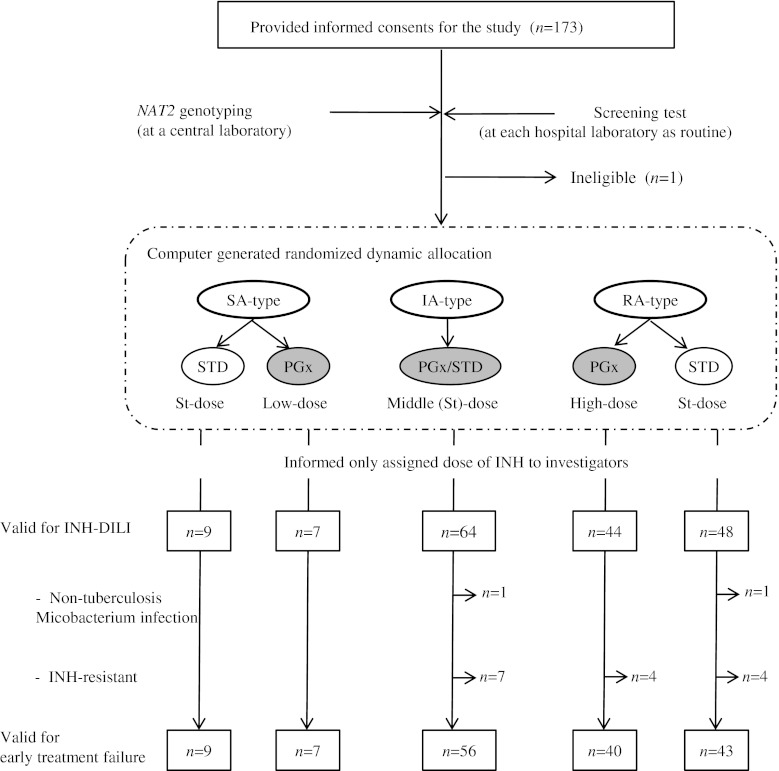
Trial profile. Abbreviations: RA-type, rapid acetylator genotype homozygous for NAT2*4; IA-type, intermediate acetylator genotypes heterozygous for NAT2*4; SA-type, slow acetylator genotypes without NAT2*4; PGx, *NAT2* genotype-guided treatment in which INH dosage was pharmacogenetically stratified based on individual NAT2 genotype; STD, conventional standard treatment with standardized dosage for all; Low-dose, half the conventional standard dose; High-dose, 1.5 times the standard dose; St-dose, conventional standard dose; INH-DILI, Drug-induced liver injury associated with isoniazid

After the allocation of the patients, *Mycobacterium avium* complex (MAC) infection and resistance to INH were examined and used as additional exclusion criteria to ensure the efficacy of INH. Patients with complex infections or INH resistance were excluded from the final analysis as illustrated in Fig. 1.

### Randomization and study medication

Before the allocation of a consenting patient, an individual *NAT2* genotype status was determined at the central laboratory and office in parallel with clinical pre-study screening at each site. Qualified patients were assigned randomly to the pharmacogenetic-guided treatment (PGx-treatment) or the conventional standard treatment (STD-treatment) according to a computer-generated dynamic randomization schedule, which minimized the imbalance of patient backgrounds including *NAT2* genotype status (rapid-, intermediate-, and slow-acetylator genotypes), study site, body weight, age, and baseline γ-glutamyltranspeptidase. Within 72 h after a blood sample was drawn for genotyping, site investigators were informed of only the dose of INH assigned to each patient (see Table 1). INH dosage for patients with more than 80 kg in body weight was not set up, in consideration for average body sizes of the Japanese population. The allocation and *NAT2* genotype status of patients were semi-concealed from all clinical staff and patients.

**Table 1:** Dose of isoniazid in *NAT2*-genotype-guided treatment or conventional standard treatment

*NAT2* status	SA-type		IA-type		RA-type	
Body weight (kg)	<40	40≤	<40	40≤	<40	40≤
Dose of isoniazid (mg, once a day)						
PGx-treatment	100	150	200	300	300	450
STD-treatment	200	300^a^	200	300^a^	200	300^a^

Isoniazid tablet (100 mg, 50 mg) was used practically. Stratified dose was 2.5, 5, 7.5 mg/kg b. w. for patients with SA-, IA-, and RA-type, respectively. SA-type, slow acetylator genotypes without NAT2*4; IA-type, intermediate acetylator genotypes heterozygous for NAT2*4; RAtype, rapid acetylator genotype homozygous for NAT2*4. PGx-treatment, *NAT2*-genotype guided treatment; STD-treatment, conventional standard treatment.

^a^Maximum dose regardless of body weight according to guidelines of ATS and JATA

In this trial, all patients were treated according to the Japan Anti-Tuberculosis Association (JATA)’s guideline with the exception of INH dosage; a 6-month regimen comprising INH, rifampicin, pyrazinamide, and ethambutol/streptomycin for the first 2 months followed by rifampicin and INH for 4 months. This treatment schedule is in line with the current standard treatment recommended internationally for adult respiratory tuberculosis [4].

Table 1 summarizes the assigned dose of INH for each parallel group. All patients started taking the standard oral dose (approx. 5 mg/kg b.w., once daily) throughout the course so as to avoid delay in initial treatment, which was the same dosing for the STD-treatment. For patients in the PGx-treatment, dosages were adjusted based on individual *NAT2* status within 3 days. Modified daily INH doses were approximately 7.5, 5 and 2.5 mg/kg for rapid, intermediate and slow acetylators, respectively. Approximately 5 mg/kg of INH, as same as the standard dose, was given to all of the intermediate acetylators throughout. Regarding the other drugs for the standard regimen, standard daily doses of rifampicin (10 mg/kg, max. 600 mg/body), pyrazinamide (25 mg/kg, 1,500 mg/body), ethambutol (15 (20) mg/kg, 750 (1,000) mg/body), and streptomycin (15 mg/kg, 750 mg/body) were recommended with the following dose ranges allowed at the discretion of the physician in charge of treatment: rifampicin, 8–12 mg/kg; pyrazinamide, 20–30 mg/kg; ethambutol, 15–20 mg/kg; streptomycin, 12–18 mg/kg. Administration of vitamin B6 was also allowed at the discretion of the physician. Primary outcomes were evaluated at the 8th week. The same treatments continued for 6 months in both groups if patients did not have any clinical need to change the ongoing treatment.

### Study procedures

The baseline clinical assessment consisted of chest radiography, demographics (age, sex, body weight, and body height), sputum examinations, and serum biochemistry tests. Three consecutive sputum samples were collected for detection of *M. tuberculosi*s which was performed by microscopy, culture and polymerase chain reaction (PCR) assay, and for susceptibility testing of *M. tuberculosis* to INH, rifampicin, ethambutol and pyrazinamide. A blood sample was drawn to determine serum concentrations of ALT, AST, ALP, bilirubin, creatinine, γ-glutamyltranspeptidase, albumin, C-reactive protein, and glucose, a full blood count, and the erythrocyte sedimentation rate. Sputum and blood samples were collected from all the patients to analyze the items above at weeks 1, 2, 3, 4, 6, and 8 during the intensive phase, and then monthly during the continuation phase for 6 months. At each point, patients were asked about any adverse events that might have occurred since the last point. Chest radiographs were taken at the 8th week, and the 24th week or the end of treatment.

### Outcome measures and assessments

The primary outcome measures were the incidences of unfavorable events in two different treatment regimens in population who required dose modification based on the *NAT2* gene polymorphism: (1) the incidences of drug–induced liver injury associated with INH (INH-DILI) that occurred within 8 weeks of the treatments, and (2) the incidences of early treatment failure at the 8th week. The secondary outcome measures were other adverse events observed during the 8-week study period.

INH-DILI was assessed according to the diagnostic criteria of the Manual for Serious Side-Effects of Drug-induced Liver Injury from the Ministry of Health, Labor and Welfare of Japan [22–24]. In brief, hepatocellular injury was defined as a more than two-fold increase in the upper limit of the normal (ULN) concentration of ALT alone or a serum ALT ratio/ALP ratio greater than 5, where the ALT ratio = ALT value/ULN of ALT, and ALP ratio = ALP value/ULN of ALP. Cholestatic injury was defined as an increase above two-fold of the upper limit of the normal range of ALP or a serum ALT ratio/ALP ratio less than 2. Mixed injury was defined as a serum ALT ratio/ALP ratio of between 2 and 5. Causality assessments showed a relationship to the INH administration if the total score was more than grade 3, i.e. ‘possible’.

Early treatment failure was defined as a combined surrogate endpoint: (1) a positive sputum culture at the 8th week examination, or (2) no sign of improvement in the chest radiograph at 8 weeks for patients with a negative sputum culture at baseline. Secondary outcome measure was defined by the other adverse events during the 8 weeks of the intensive phase of the anti-tuberculosis therapy.

All outcomes were determined by the members of a separate central end point adjudication committee who used standard definitions and were unaware of treatment allocation and *NAT2* genotypes. An independent Data and Safety Monitoring Committee could advise early termination of the trial for safety, scientific or ethical reasons.

### NAT2 genotyping

Genomic DNA was extracted from peripheral whole blood of each subject using a QIA amp Blood Mini Kit (QIAGEN). Six single nucleotide polymorphisms in the *NAT2* gene were analyzed: 190C to T (NAT2*19), 191 G to A (NAT2*14), 341 T to C (NAT2*5), 481C to T, 590 G to A (NAT2*6), and 857 G to A (NAT2*7). Detections were separately duplicated for quality control of genotyping. In brief, the three mutations 481C to T, 590 G to A and 857 G to A were detected by a PCR-RFLP (restriction fragment length polymorphism) method as described previously [17]. After PCR amplification using specific primers, the PCR products were digested with the restriction enzymes *Kpn* I, *Taq* I, and *Bam* HI to determine the nucleotide substitution at 481, 590, and 857, respectively. A mismatch PCR-RFLP procedure was performed to detect the 341 T to C, 190C to T and 191 G to A substitutions, as described previously [20]. The restriction enzymes *Dde* I and *Msp* I were used for the PCR products to determine the nucleotide substitutions at 341 and 190/191, respectively. Alleles without any defective forms were defined as functional allele, NAT2*4.

### Sample size calculation

On the basis of data from our previous study [18, 25], we assumed that the incidence of INH-induced liver injury in slow acetylators would be 65 % in the control arm (STD-treatment; conventional standard dosing of INH) and 15 % in the test arm (PGx-treatment; *NAT2* genotype-guided dosing of INH). We hypothesized that the occurrence of early therapeutic failure in rapid acetylators would be decreased, from 35 % in the STD-treatment to 15 % in the PGx-treatment [26]. To achieve a power of 0.8 with an alpha of 0.05, we aimed to enroll 22 slow acetylators and 156 evaluable rapid acetylators in the trial. According to our calculations, a maximum sample size of 360 patients would be needed to test this hypothesis, allowing for a 15 % dropout rate, with frequencies of rapid, intermediate and slow acetylators estimated at 0.50, 0.42 and 0.08, respectively. A group sequential analysis would be performed if 16 slow acetylators were assessable. The enrollment would be stopped when *p* values regarding INH-DILI in slow acetylators were less than O’Brien-Fleming type stopping boundary determined by Lan-DeMets approach. Then, the trial would be finalized early because of ethical considerations.

### Statistical analysis

The analyses were based on the intention-to-treat approach, including all assessable randomized patients. Since the patients with intermediate genotypes receive the same dose of INH irrespective of the results of the randomization, their data were analyzed as a combined group. A two-tailed significance test was used. Categorical data were compared using the *χ*2 test or Fisher’s exact test as appropriate and Cochran-Mantel-Haenszel test was used for multivariate analyses. Continuous variables are presented as the mean and standard deviation (SD) or 25 %, 50 % and 75 % percentiles as applicable and were analyzed by ANOVA with posthoc and the Student-*t* test (for parametric variables) and the Mann-Whitney *U* test (for non-parametric variables). The Lan-DeMets alpha spending function was employed for major outcomes. Multivariable logistic analyses were performed to evaluate the relationship between the incidence of unfavorable events and the baseline factors/covariates. Analyses were performed using SPSS ver. 11.0 (SPSS Inc.) and R ver. 2.10.1 (The R Foundation for Statistical Computing).

## Results

### Patient enrollment and demographics

In all, 172 patients were randomly assigned to one of the two study regimens, the *NAT2* genotype-guided dosing treatment (PGx-treatment) and the conventional standard dosing treatment (STD-treatment), as summarized in Fig. 1. The patients’ backgrounds showed a balanced distribution (Table 2). Eighty three patients were assigned to the PGx-guided therapy and 89 to the empirical therapy. Among them, 17 patients were not eligible for analysis since they met exclusion criteria after their allocation; 12 with primary INH-resistant tuberculosis and five with MAC infections. The rest of the 155 patients were infected with organisms sensitive to all first-line anti-TB drugs. The distribution of *NAT2* genotype frequencies was similar to that in our previous study [18].

**Table 2:** Baseline characteristics of all randomized participants valid for evaluation of isoniazid-related liver injury and other adverse events on intention-to-treat bases

Parameters	NAT2 genotype status	SA-type		IA-type	RA-type		*p* value
	Treatments	STD	PGx	PGx/STD	PGx	STD	
Patients (*n*)		9	7	64	44	48	
Sex (M/F, *n*)		4/5	4/3	44/20	25/19	35/13	
Age (years)^a^		35.0	48.0	48.5	50.5	51.0	0.614
		(35.0–43.0)	(36.0–58.0)	(34.0, 60.0)	(33.0, 57.8)	(30.0, 60.2)	
Weight (kg)^a^		55.0	59.5	54.1	50.0	57.5	0.058
		(52.8–59.8)	(47.9–62.8)	(50.1–62.2)	(45.9–55.2)	(47.6–63.8)	
AST (IU/L)^a^		18	17	18	16	19	0.165
		(15–22)	(17–27)	(15–24)	(13–20)	(15–22)	
ALT (IU/L)^a^		12	15	14	12	15	0.066
		(10–19)	(9–24)	(10–21)	(8–16)	(11–21)	
ALP (IU/L)^a^		241	244	238	255	242	0.696
		(214–305)	(195–329)	(184–285)	(208–304)	(205–288)	
Total-bilirubin (mg/dL)^a^		0.3	0.5	0.6	0.5	0.6	0.338
		(0.3–0.6)	(0.5–0.8)	(0.4–0.7)	(0.4–0.6)	(0.4–0.7)	
Creatinine (mg/dL)^a^		0.6	0.6	0.7	0.7	0.7	0.462
		(0.5–0.7)	(0.5–0.8)	(0.6–0.8)	(0.5–0.8)	(0.6–0.8)	
Anti-tuberculosis drugs (Dose, mg/kg/day)^b^							
Isoniazid^b^		5.5 (0.8)	2.6 (0.3)	5.5 (0.9)	8.8 (1.3)	5.5 (1.0)	NA
Rifampicin^b^		8.7 (1.4)	8.9 (1.7)	8.7 (1.4)	9.5 (1.4)	9.4 (1.5)	0.076
Pyrazinamide^b^		23.7 (3.0)	20.7 (4.3)	22.1 (5.9)	22.7 (2.7)	22.8 (4.6)	0.676
Ethambutol^b^		14.5 (2.0)	15.4 (3.2)	14.4 (3.5)	15.4 (2.5)	15.3 (4.1)	0.555
Concomitant drug (%)							
Vitamin B6		44.4	50.0	40.6	43.2	43.8	
Primary sputum culture (*n*) of complex infection or INH resistance							
Negative		1	0	21	8	8	
Positive-low (< +++)		7	6	26	22	26	
Positive-high (> = +++)		1	1	9	9	10	

^a^Data are presented as the median (25 %, 75 %).

^b^Data are presented as means (SD).

There was no statistical difference between PGx and STD in IA. Five groups were compared by ANOVA. PGx, NAT2 genotype-guided treatment group; STD, conventional standard treatment group; RA-type, rapid acetylator genotype homozygous for NAT2*4; IA-type, intermediate acetylator genotypes heterozygous for NAT2*4; SA-type, slow acetylator genotypes without NAT2*4. NA, not applied

### Primary Outcomes

#### Drug-induced liver injury associated with isoniazid (INH-DILI)

Seven of nine slow acetylators in the STD-treatment experienced INH-related liver injury (INH-DILI) within the first 4 weeks, while none of the seven slow acetylators in the PGx-treatment did during the first 8 weeks (attribute risk difference; 77.8 %, *p* = 0.003, Table 3a). Conversely, few rapid acetylators in either group had INH-DILI during the first 8 weeks; 4.2 % (2/48 patients) in the STD-treatment and 4.6 % (2/44 patients) in the PGx-treatment receiving high-dose INH, which were importantly equivalent to the incidence in intermediate acetylators (4.7 %, 3/64 patients). These outcomes were not different in a population valid for early treatment failure. Multivariable logistic analysis using the baseline patient characteristics revealed that the NAT2 slow acetylator genotype was associated with INH-DILI in the STD-treatment, but not in the PGx-treatment at all, suggesting standard dose of INH is too much for slow acetylators. There was not any other factors and confounders associated with INH-DILI.

**Table 3:** Primary end point results in slow and rapid acetylator genotypes

*NAT2* genotype status	Groups	(n)	Incidence (%)	Univariate			Multivariate ^e^		
				*p* values	Relative risk (95 % CI)		*p* values	Pooled odds ratios (95 % CI)	
a) Isoniazid-related liver injury									
SA-type	PGx	(7)	0.0	0.003^a^	–		NA^d^		
	STD	(9)	77.8						
RA-type^c^	PGx	(44)	4.5	1.000	1.091	(0.160–7.42)	NA^d^		
	STD	(48)	4.2	reference					
b) Early treatment failure									
SA-type ^c^	PGx	(7)	0.0	0.475	–	–	0.595	–	–
	STD	(9)	22.2						
RA-type	PGx	(40)	15.0	0.013^b^	0.379	(0.166–0.866)	0.015^b^	0.274	(0.097–0.776)
	STD	(43)	39.5	reference			reference		
c) Unfavorable event, i.e., combined endpoints of isoniazid-related liver injury or early treatment failure									
RA+SA-type	PGx	(47)	17.0	0.001^a^	0.354	(0.177–0.707)	0.002^a^	0.229	(0.091–0.574)
	STD	(52)	48.1	reference			reference		

PGx=NAT2 genotype-guided dosing group, STD=Conventional standard dosing group

^a^less than O’Brien-Fleming type stopping boundaries of the Lan-DeMets alpha spending function

^b^less than Pocock type stopping boundaries

^c^secondary end point of each NAT2 genotype status

^d^not applied because of no confounder, and

^d^Cochrane–Mantel–Haenszel test stratified by high-excretion quantity level of tubercle bacilli of sputum culture at the screening test

#### Early treatment failure

The therapeutic effects of *NAT2* genotype-guided dosing on early treatment failure against drug-sensitive tuberculosis are shown in Table 3b). The *NAT2* genotype-guided regimen successfully reduced the incidence of early treatment failure in rapid acetylators; 15.0 % (6/40 patients) in the PGx-treatment and 39.5 % (17/43 patients) in the STD-treatment (relative risk; 0.379 [95 % CI: 0.116, 0.866], *p* = 0.013). The difference was distinct on multivariate analysis stratified by high-excretion level of tubercle bacilli (Pooled odds ratio; 0.274 [95 % CI: 0.097, 0.776], *p* = 0.015). One rapid acetylator in the STD-treatment who developed INH-DILI failed to achieve any clinical improvement by the 8th week of treatment. Importantly, two of nine (22.2 %) slow acetylators with INH-DILI in the STD-treatment (5 mg/kg) did not show any clinical improvement. In these cases, their standard treatment was interrupted due to the INH-DILI and it was inevitable to withdraw INH for one of them. In contrast, all the slow acetylators in the PGx-treatment achieved 8 weeks of treatment successfully, even with a lower dose of isoniazid than in convention therapy.

#### Combined unfavorable events

The incidence of combined unfavorable events, defined by early therapeutic failure or INH-related drug-induced liver injury, was significantly lower in the combined population of rapid and slow acetylators; 17.0 % in the PGx-treatment and 48.1 % in the STD-treatment (relative risk; 0.354 [95%CI: 0.177, 0.707], *p* = 0.001 (Table 3c)). Furthermore, the risk reduction was more conspicuous when the subjects were stratified by the level of primary sputum culture; pooled odds ratios [95%CI] were 0.229 [0.091, 0.548] in combined population of rapid and slow acetylators.

**Fig. 2:**
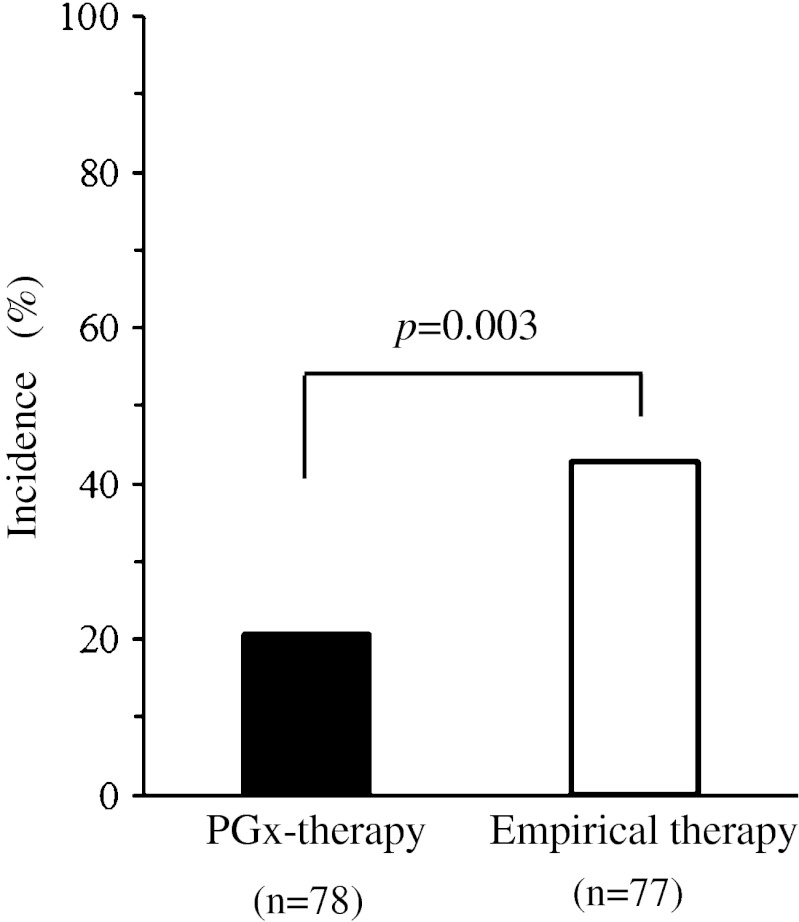
Overall incidence of combined unfavorable events in the patients who received PGx-therapy and in those who received empirical therapy

Although the dose of INH for intermediate acetylators in the PGx-treatment was the same as that in the STD-treatment, overall clinical usefulness of the PGx-guided therapy was compared with the empirical therapy: the combined unfavorable events were 20.5 % (16/78 patients) in PGx-guided therapy and 42.9 % (33/77 patients) in the empirical therapy (relative risk; 0.479 [95%CI: 0.288, 0.795], *p* = 0.003) (Fig. 2).

#### Sub-analyses

**Fig. 3:**
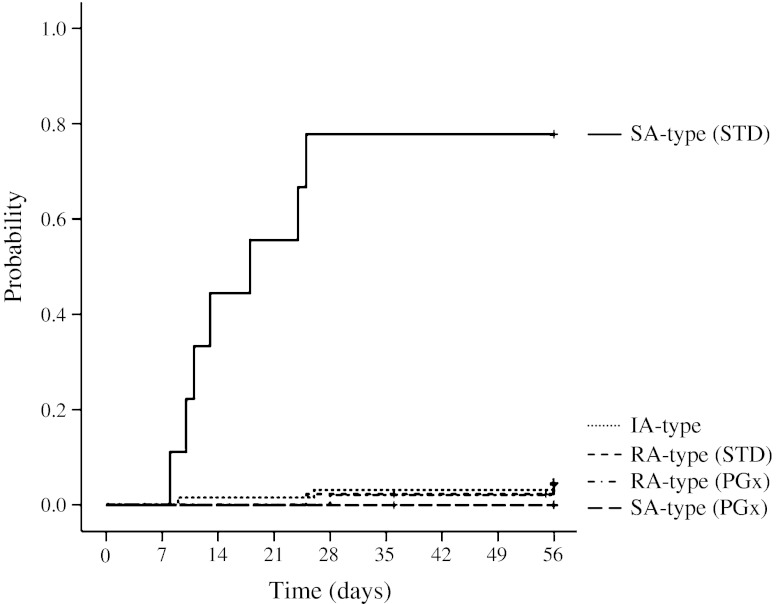
Cumulative incidence curves of isoniazid related drug-induced liver injury (INH-DILI) over time among the 172 patients. RA-type, rapid acetylator genotype; IA-type, intermediate acetylator genotypes; SA-type, slow acetylator genotypes; PGx, PGx-guided treatment; STD, conventional standard-treatment

The cumulative incidence of INH-DILI over time is illustrated in Fig. 3. Mean (SD) time to detect INH-DILI was 15.6 (6.9) days in slow acetylators receiving the conventional standard treatment. As for intermediate acetylators, INH-DILI, early therapeutic failure and the combined unfavorable events occurred in 4.7 % (3/64 patients), 26.8 % (15/56 patients) and 28.6 % (16/56 patients), respectively. Overall probabilities of the combined unfavorable events were 23.3 % (24/103 patients) in the PGx-treatment (8/47 patients) plus intermediate acetylators (16/56 patients) and 48.1 % (25/52 patients) in the STD-treatment.

**Fig. 4:**
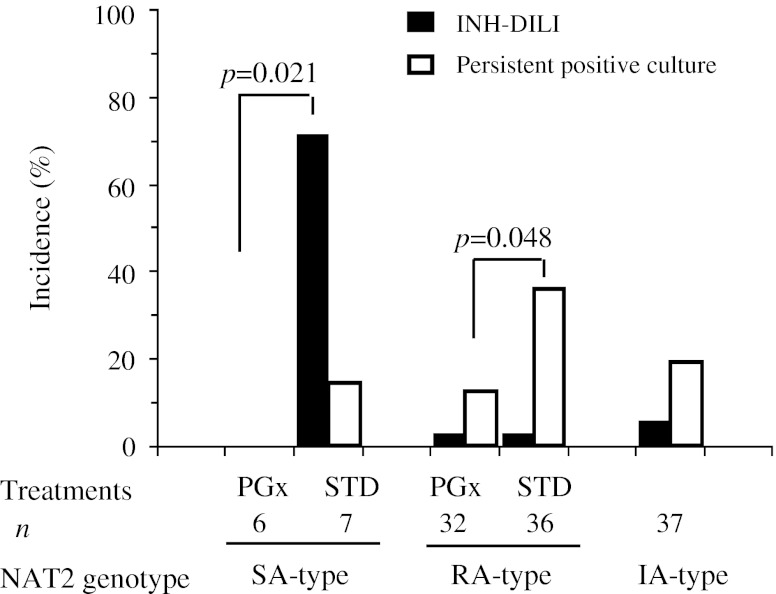
Incidence of isoniazid induced liver injury (INH-DILI) and persistent positive culture among the patients with drug sensitive tuberculosis on sputum culture at screening. Closed columns, INH-DILI; open columns, persistent positive culture; RA-type, rapid acetylator genotype; IA-type, intermediate acetylator genotypes; SA-type, slow acetylator genotypes; PGx, PGx-guided treatment; STD, conventional standard-treatment

**Fig. 5:**
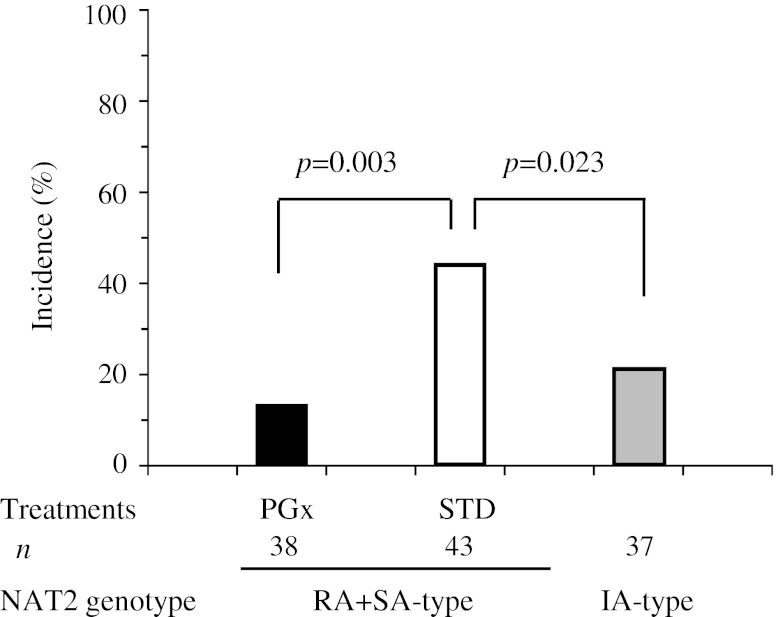
Incidence of combined unfavorable events among the patients with drug sensitive tuberculosis on sputum culture at screening. RA + IA-type, combined with rapid or slow acetylator genotypes; IA-type, intermediate acetylator genotypes; PGx, PGx-guided treatment; STD, conventional standard-treatment

Further explorative analyses were performed in 118 patients presenting as sputum culture-positive at screening, who were infected with *M. tuberculosis* sensitive to first-line anti-tuberculosis drugs. As well as primary outcomes, PGx-guided treatment reduced INH-DILI with keeping enough therapeutic efficacies in slow acetylators, and also reduced incidence of persistent positive culture without increase of INH-DILI frequency in rapid acetylators (Fig. 4). Regarding persistent positive sputum culture at the 8th week, the risk difference was striking in rapid acetylators with highly-positive level of organisms on primary sputum culture; 10.0 % (1/10 patients) in the PGx-treatment and 66.7 % (6/9 patients) in the STD-treatment (*p* = 0.020). Incidence of unfavorable events of the combined population of rapid and slow acetylators was obviously higher in the STD-treatment than in PGx-treatment. In contrast, the PGx-guided dose stratification reduced unfavorable events to the level of intermediate acetylators’ (Fig. 5).

#### Secondary outcomes

Other adverse events observed during the 8-week study period were skin and subcutaneous tissue disorders including rashes (n = 48), nervous system disorders including headaches (n = 22), gastrointestinal disorders (n = 22), peripheral neuropathy (n = 13), visual impediment (n = 3), and hearing impairment (n = 3). Peripheral neuropathy was observed in two of nine slow acetylators in the STD-treatment, but not at all in the PGx-treatment (22 % vs 0 %). As for the other genotypes, peripheral neuropathy was observed in four of 48 patients who received the STD-treatment and three of 44 patients who received the PGx-treatment (8 % vs 7 %) in the group of rapid acetylators, and four of 64 (6 %) in the group of intermediate acetylators. Among the 13 patients presenting with this event, seven patients received vitamin B6: one slow acetylator and three rapid acetylators in the STD-treatment, one rapid acetylator in the PGx-treatment, and three intermediate acetylators. There was no statistically significant difference in the incidence of these adverse events between the PGx-treatment group and STD-treatment group.

## Discussion

In the present study we have demonstrated the significant therapeutic potential of the *NAT2* genotype-guided dosing of INH in the intensive phase of the internationally standardized 6-month four-drug regimen for newly diagnosed pulmonary tuberculosis. The *new* dosing regimen significantly reduced the incidences of INH-DILI and early treatment failure, compared to the conventional standardized regimen. The slow acetylators assigned to the PGx-treatment, who were administered a lower dose of INH than the STD-treatment, developed no INH-related liver injury, but achieved clinical improvements within 8 weeks. On the other hand, the rapid acetylators in the PGx-treatment, receiving a higher dose of INH, achieved a 25 % higher rate of clinical improvement by the 8th week than those in the STD-treatment, with an incidence of INH-related liver injury of less than 5 %. The absolute risk reduction in the combined population of rapid and slow acetylators was 31 % with respect to unfavorable events, i.e., INH-DILI or early treatment failure.

*NAT2* genotypic status has been focused on as a genomic biomarker for individualized medicine to preempt INH-related unfavorable events. In fact, INH-induced hepatotoxicity has been studied enthusiastically worldwide since we reported its association with slow acetylator genotypes [18]. In accordance with previous observational studies [18, 19], the present randomized clinical trial has confirmed that slow acetylators are prone to INH-DILI by conventional standard treatment with the relative risk of 4.5 (95 % CI, 1.3–15.3). Importantly, the present trial has clearly demonstrated that the incidence of INH-DILI was strikingly decreased by the *NAT2*-guided dosing stratification of INH. These results suggest that *NAT2*-guided dosing for slow acetylators would contribute to the preemptive discontinuation of anti-tuberculous drugs, thereby reducing the incidence of treatment failure and/or relapse of tuberculosis.

It has also been discussed that rapid acetylators are prone to treatment failure during anti-tuberculosis therapy based on INH [13, 15, 16]. Consistent with this, we showed that the risk of treatment failure was higher in genotypic rapid acetylators, treated with the conventional dosage of INH, in the present study. Accumulating evidence has revealed the mechanisms of treatment failure with the four-drug standard regimen. Patients with low INH plasma concentrations have a propensity for treatment failure or early relapse of tuberculosis [27–29]. Indeed, in vitro and in vivo animal studies have demonstrated that the cumulative antibacterial effect of INH is associated with the area under the INH concentration versus time curve [30]. In combination therapy for tuberculosis, the main population of *M. tuberculosis* is susceptible to INH, while pyrazinamide is effective against slower growing bacilli in acidic milieus, and rifampicin against nonreplicating bacilli. During the first 2 to 5 days of the multidrug therapy, the bactericidal activity is derived mainly from INH [31, 32]. This bactericidal activity ceases due to the emergence of an INH-resistant population [33], resulting in treatment failure. In the present study, NAT2 genotype-guided dosing stratification of INH obviously improves therapeutic efficacy against INH-sensitive tuberculosis during the first 8 weeks, although the PGx-treatment could not start until a couple of days after the first dose. This fact strongly indicates that appropriate dose adjustment of INH at the beginning of therapy is important to avoid treatment failure and the relapse of tuberculosis, especially for patients infected with *M. tubeculusosis* sensitive to INH.

Of note, the success rate for anti-tuberculosis treatment has been lower in Japan than in Western countries [1, 34]. Although a Japanese authority, JATA, has been propelling WHO-recommended programs, and has changed the standard INH dose from 400–450 mg to 300 mg, the treatment–success rate has not been improved in Japan. Global Health Observatory Data reported that smear-positive tuberculosis treatment-success rates were 52 % in Japan and 69–95 % in Europe in 2009 [1]. The lower success rate in the Japanese population might be explained by an interethnic difference in *NAT2* genotype frequencies. The frequency of rapid acetylator genotypes in the Japanese population (approx. 50 %) is much higher than that in Caucasians (approx. 5 %) [12, 18, 35, 36]. Taken together, the dose of INH used in conventional treatment is insufficient for more than a third of Japanese with NAT2*4/*4 in achieving a satisfactory treatment-success rate. *NAT2* genotype-guided dosing stratification would improve early treatment success rates against tuberculosis, especially in highly-positive excretion levels of bacilli, if the high dose of INH was applied from the first prescription exclusively to rapid acetylators.

In conclusion, the present pharmacogenetics clinical trial with a PROBE-design demonstrated that *NAT2* genotype-guided dosing stratification of INH could improve treatment outcomes in patients with drug-sensitive tuberculosis. Half of the conventional standard dose for slow acetylators reduced INH-DILI and did not reduce therapeutic efficacy. For rapid acetylators, 1.5 times the conventional standard dose of INH reduced early treatment failures without an increase in the incidence of INH-DILI. We propose here that the pharmacotherapy against tuberculosis with INH should be individually designed by stratification based on *NAT2* genetic information in order to achieve the greatest success outcome for each patient.

## References

[1] Global Health Observatory (GHO); Tuberculosis (TB). World Health Organization Web site. http://www.who.int/gho/tb/en/index.html Accessed Sep 10, 2012.

[2] The Global Plan to Stop TB. Stop TB Partnership Web site. http://www.stoptb.org/global/plan/ Accessed Sep 10, 2012.

[3] Guidelines for treatment of tuberculosis, 4th ed. World Health Organization Web site. http://www.who.int/tb/publications/2010/9789241547833/en/ Accessed Sep 10, 2012.

[4] Blumberg HM, Burman WJ, Chaisson RE, Daley CL, Etkind SC, Friedman LN, Fujiwara P, Grzemska M, Hopewell PC, Iseman MD, Jasmer RM, Koppaka V, Menzies RI, O’Brien RJ, Reves RR, Reichman LB, Simone PM, Starke JR, Vernon AA (2003). American Thoracic Society/Centers for Disease Control and Prevention/Infectious Diseases Society of America: treatment of tuberculosis. Am J Respir Crit Care Med.

[5] Yee D, Valiquette C, Pelletier M, Parisien I, Rocher I, Menzies D (2003). Incidence of serious side effects from first-line antituberculosis drugs among patients treated for active tuberculosis. Am J Respir Crit Care Med.

[6] Steele MA, Burk RF, DeaPrez RM (1991). Toxic hepatitis with isoniazid and rifampin. A meta-analysis. Chest.

[7] Jindani A, Nunn AJ, Enarson DA (2004). Two 8-month regimens of chemotherapy for treatment of newly diagnosed pulmonary tuberculosis: international multicentre randomised trial. Lancet.

[8] Durand F, Jebrak G, Pessayre D, Fournier M, Bernuau J (1996). Hepatotoxicity of antitubercular treatments. Rationale for monitoring liver status. Drug Saf.

[9] Evans DA, Manley KA, Mc KV (1960). Genetic control of isoniazid metabolism in man. Br Med J.

[10] Evans DA, Storey PB, Wittstadt FB, Manley KA (1960). The determination of the isoniazid inactivator phenotype. Am Rev Respir Dis.

[11] Human NAT2 alleles (Haplotypes) (updated July 22, 2011) http://louisville.edu/medschool/pharmacology/consensus-human-arylamine-n-acetyltransferase-gene-nomenclature/nat_pdf_files/Human_NAT2_alleles.pdf Accessed Sep 10, 2012.

[12] Mashimo M, Suzuki T, Abe M, Deguchi T (1992). Molecular genotyping of N-acetylation polymorphism to predict phenotype. Hum Genet.

[13] Parkin DP, Vandenplas S, Botha FJ, Vandenplas ML, Seifart HI, van Helden PD, van der Walt BJ, Donald PR, van Jaarsveld PP (1997). Trimodality of isoniazid elimination: phenotype and genotype in patients with tuberculosis. Am J Respir Crit Care Med.

[14] Ellard GA (1976). Variations between individuals and populations in the acetylation of isoniazid and its significance for the treatment of pulmonary tuberculosis. Clin Pharmacol Ther.

[15] Donald PR, Sirgel FA, Venter A, Parkin DP, Seifart HI, van de Wal BW, Werely C, van Helden PD, Maritz JS (2004). The influence of human N-acetyltransferase genotype on the early bactericidal activity of isoniazid. Clin Infect Dis.

[16] Kubota R, Ohno M, Yasunaga M, Yokota S, Maekura R, Azuma J (2005). Tentative treatments for tuberculosis based on N-acetyltransferase gene polymorphism. Jpn J Therapeutic Drug Monitoring.

[17] Evans DA (1989). N-acetyltransferase. Pharmacol Ther.

[18] Ohno M, Yamaguchi I, Yamamoto I, Fukuda T, Yokota S, Maekura R, Ito M, Yamamoto Y, Ogura T, Maeda K (2000). Slow N-acetyltransferase 2 genotype affects the incidence of isoniazid and rifampicin-induced hepatotoxicity. Int J Tuberc Lung Dis.

[19] Huang YS, Chern HD, Su WJ, Wu JC, Lai SL, Yang SY, Chang FY, Lee SD (2002). Polymorphism of the N-acetyltransferase 2 gene as a susceptibility risk factor for antituberculosis drug-induced hepatitis. Hepatology.

[20] Kinzig-Schippers M, Tomalik-Scharte D, Jetter A, Scheidel B, Jakob V, Rodamer M, Cascorbi I, Doroshyenko O, Sorgel F, Fuhr U (2005). Should we use N-acetyltransferase type 2 genotyping to personalize isoniazid doses?. Antimicrob Agents Chemother.

[21] Kubota R, Ohno M, Hasunuma T, Iijima H, Azuma J (2007). Dose-escalation study of isoniazid in healthy volunteers with the rapid acetylator genotype of arylamine *N*-acetyltransferase 2. Eur J Clin Pharmacol.

[22] http://www.info.pmda.go.jp/juutoku/file/jfm0804002.pdf Accessed Sep 10, 2012.

[23] Takikawa H, Onji M (2005). A proposal of the diagnostic criteria of drug-induced liver injury. Hepatol Res.

[24] Danan G, Benichou C (1993). Causality assessment of adverse reactions to drugs. A novel method based on the conclusions of international consensus meetings: Application to drug-Induced liver injuries. J Clin Epidemiol.

[25] Tokuda A, Ohno M, Furutsuka M, Kubota R, Nakayama S, Yokota S, Maekura R, Azuma J (2006). GSTs and NAT2 gene polymorphisms and drug induced hepatotoxicity in anti-tuberculosis therapy. Jpn J Clin Pharmacol Ther.

[26] Mitchison DA (1993). Assessment of new sterilizing drugs for treating pulmonary tuberculosis by culture at 2 months. Am Rev Respir Dis.

[27] Kimerling ME, Phillips P, Patterson P, Hall M, Robinson CA, Dunlap NE (1998). Low serum antimycobacterial drug levels in non-HIV-infected tuberculosis patients. Chest.

[28] Weiner M, Burman W, Vernon A, Benator D, Peloquin CA, Khan A, Weis S, King B, Shah N, Hodge T (2003). Low isoniazid concentrations and outcome of tuberculosis treatment with once-weekly isoniazid and rifapentine. Am J Respir Crit Care Med.

[29] Weiner M, Benator D, Burman W, Peloquin CA, Khan A, Vernon A, Jones B, Silva-Trigo C, Zhao Z, Hodge T (2005). Association between acquired rifamycin resistance and the pharmacokinetics of rifabutin and isoniazid among patients with HIV and tuberculosis. Clin Infect Dis.

[30] Jayaram R, Shandil RK, Gaonkar S, Kaur P, Suresh BL, Mahesh BN, Jayashree R, Nandi V, Bharath S, Kantharaj E, Balasubramanian V (2004). Isoniazid pharmacokinetics-pharmacodynamics in an aerosol infection model of tuberculosis. Antimicrob Agents Chemother.

[31] Jindani A, Aber VR, Edwards EA, and Mitchison DA (1980) The early bactericidal activity of drugs in patients with pulmonary tuberculosis. Am Rev Respir Dis 121-939-949. 10.1164/arrd.1980.121.6.939 6774638

[32] Jindani A, Cj D, Mitchison DA (2003). Bactericidal and sterilizing activities of antituberculosis drugs during the first 14 days. Am J Resp Crit Care Med.

[33] Gumbo T, Louie A, Liu W, Ambrose PG, Bhavnani SM, Brown D, Drusano GL (2007). Isoniazid’s bactericidal activity ceases because of the emergence of resistance, not depletion of *Mycobacterium tuberculosis* in the log phase of growth. J Infect Dis.

[34] Abe C, Hirano K, Wada M, Aoyagi T (2001). Resistance of Mycobacterium tuberculosis of four first-line anti-tuberculosis drugs in Japan, 1997. Int J Tuberc Lung Dis.

[35] Sunahara S, Urano M, Ogawa M (1961). Genetical and geographic studies on isoniazid inactivation. Science.

[36] Cascorbi I, Drakoulis N, Brockmoller J, Maurer A, Sperling K, Roots I (1995). Arylamine N-acetyltransferase (NAT2) mutations and their allelic linkage in unrelated Caucasian individuals: correlation with phenotypic activity. Am J Hum Genet.

